# Protracted Development on Native Tone Interpretation: Evidence From Mandarin-Learning Infants’ Novel Word Learning

**DOI:** 10.3389/fpsyg.2019.01512

**Published:** 2019-07-05

**Authors:** Xiaobei Zheng, Yinglin Ji, Xiangzhi Meng

**Affiliations:** ^1^Research Centre for Language and Cognition, School of Foreign Languages, Shenzhen University, Shenzhen, China; ^2^Research Centre for Language and Cognition, School of Arts and Humanities, Shenzhen University, Shenzhen, China; ^3^Beijing Key Laboratory of Behavior and Mental Health, School of Psychological and Cognitive Sciences, Peking University, Beijing, China

**Keywords:** infant word learning, Mandarin tone, habituation, word-object association, tone, intonation

## Abstract

Studies have shown that infants from cultures with tone languages develop categorical perception of their native lexical tone before their first birthday, but few studies have explored whether, and when, they interpret the phonemic function of lexical tone in word learning. Two habituation-switch experiments were conducted to explore whether Mandarin-learning infants could exploit tonal cues during their word learning, and detect a change when the association of two word-object pairs was switched. In Experiment 1, two words were solely differentiated by their lexical tones (/fāi/ vs. /făi/), and Mandarin-learning infants failed to detect the switch of tones at 14 months, but succeeded at 18 months. In Experiment 2, two words were markedly distinct (/fāi/ vs. /bǒu/), and infants could detect the change of words as early as 14 months. The results indicate that infants may not refer to the lexical function of tone during their novel word learning until 18 months, even though infants from birth are able to distinguish the Tone 1 vs. Tone 3 contrast. Given that lexical tone is expressed by variations of the pitch contours, which are also related to intonation, infants’ increasing knowledge of both tone and intonation may contribute to their misinterpretation of pitch contours in word learning at 14 months and, further, to their development of a sophisticated use of the phonemic function of lexical tone at 18 months of age.

## Introduction

**Figure 1:**
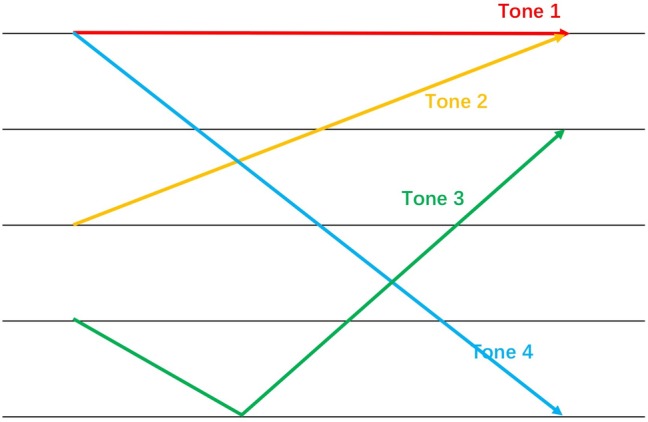
The sample pitch contours of four Mandarin tones.

Seventy percent of languages globally are classified as tonal (Yip, 2002) and are spoken by more than 50% of the world’s population (Fromkin, 1978). Mandarin is one of the most prevalent tonal languages. Tones are defined by fundamental frequency (f0) characteristics. F0 is a sound property perceived as pitch. There are two types of f0 tracks: static level tones (i.e., high-, middle-, and low-level tones) and dynamic contour tones (i.e., rising and falling tones) within single syllables (Vance, 1976; Gandour and Harshman, 1978; Gandour, 1981; Khouw and Ciocca, 2007). Mandarin lexical tones are categorized as Tone 1 (high-level), Tone 2 (rising), Tone 3 (falling then rising), and Tone 4 (falling) (see Figure 1). Tone 1 is a static tone, and the other three are dynamic. Different tones create completely different meanings for the same syllable. Examples include /mā/, “*mother*,” vs. /mă/, “*horse*,” and /shū/, “*book*,” vs. /shù/, “tree.” Therefore, tone perception is fundamental to Mandarin word acquisition.

The development of lexical tones is complicated: it involves the perception of pitch, which is also used to convey paralanguage information in intonation. Pitch is embedded in the Mandarin language environment in two ways. Firstly, lexical tones designate and discriminate lexical meaning and, secondly, intonations encode prosodic information. Just as consonants and vowels change the lexical meaning of words and concepts in other languages, different tones, expressed by variations of the pitch contour, can change the lexical meaning in Mandarin. At the same time, intonations, which are also expressed by pitch contours, are supra-segmental units that convey emotional or pragmatic prosody (Singh and Fu, 2016). This means that the lexical and prosodic meanings are in competition when a tone shares the same pitch contour as an intonation. This raises two questions for exploration: how do infants from tonal language-speaking cultures perceive their native tonal contrasts? And how do they interpret these tonal contrasts as lexical cues during word learning?

### Language-Specific Tone Perception

Studies have shown that infants can perceive tonal contrasts at an early age. The acoustic property of f0 variations is perceptually salient enough to reach the fetal auditory system (Lecanuet, 1998; Mampe et al., 2009). For example, French-born neonates can distinguish high-low vs. low-high pitch levels for 24 bisyllabic Japanese word samples (Nazzi et al., 1998). Neonates of Mandarin Chinese also show neural responses to the changes in the Mandarin Tone 1 vs. Tone 3 pair, and the Tone 2 vs. Tone 3 pair, in an event-related potential study (Cheng et al., 2013). Older American-born infants, aged 2–3 months, can distinguish synthesized [ra] and [la] tokens with rising vs. falling pitch contours (Karzon and Nicholas, 1989).

Previous research has revealed that tonal language-learning infants maintain and increase their sensitivity to native tonal contrasts, whereas the sensitivity to tonal contrasts declines in non-tonal language-learning infants during the latter half of their first year. Mandarin-learning infants can discriminate Thai tone contrasts (e.g., a rising vs. a low-level tone) at 6 months of age and they maintain this discrimination in the following 3 months. In contrast, their English counterparts demonstrate a decline in their discrimination performance from 6 to 9 months (Mattock and Burnham, 2006). Similarly, stable tone discrimination was reported in Chinese infants from 4 to 9 months of age, while a decline in tone discrimination was reported in English infants during the same period (Yeung et al., 2013).

In the second year of their life, even though tonal language-learning infants maintain their sensitivity to native tonal contrasts, non-tonal language learners demonstrate a rebound of tonal perception. Dutch-exposed infants demonstrate a discrimination of Mandarin tone contrasts only during two specific periods of age: 5–6 and 17–18 months, but a decline in tone sensitivity between 5 and 18 months (Liu and Kager, 2014). German-learning infants, who discriminate Cantonese lexical tones at 6 and 18 months of age, fail at 9 months (Götz et al., 2018). Generally, non-tonal language learners demonstrate a U-shape development: a perceptual decrease followed by a rebound during the first 2 years of age, in tonal contrasts.

In addition to testing the general developmental trajectory of tone discrimination, studies have examined the perception of individual tonal contrasts in tonal language learners. It is widely acknowledged that the Mandarin Tone 1 and Tone 3 pair is the most distinctive tonal contrast (Cheng et al., 2013; Ma et al., 2017; Tsao, 2017). Tone 3 experiences a double contour (falling then rising), so it significantly differs from Tone 1 (the static tone). In contrast, any pairs of the other three contour tones (Tone 2, 3, and 4) are similar to some extent because they all involve pitch contours (see Figure 1). In other words, the static vs. dynamic contrast is more salient than the dynamic vs. dynamic contrast. For example, the static (Tone 1) vs. dynamic (Tone 3) pair elicits an auditory change-related cortical response in newborn Mandarin-learning infants, while the Tone 2 vs. Tone 3 pair only elicits the response in the 6-month olds (Cheng et al., 2013). Furthermore, the ability to discriminate the Tone 1 and Tone 3 contrast significantly accelerates infants’ development of the perception of this tonal contrast as compared to the dynamic vs. dynamic pairs. Mandarin-learning infants’ perception of the static (Tone 1) vs. dynamic (Tone 3) contrast increases from 6 to 12 months, while the perception of the dynamic vs. dynamic contrasts (e.g., the Tone 2 vs. Tone 3 pair and the Tone 2 vs. Tone 4 pair) remains the same during this period (Tsao, 2017). In sum, both behavioral and neurological studies have suggested that the Tone 1 vs. Tone 3 pair is more perceptually distinctive than any of the dynamic vs. dynamic contrasts (i.e., the tonal combinations of any two of Tone 2, Tone 3 and Tone 4) for Mandarin-learning infants (Table 1)^1^.

**Table 1:** A summary of studies on Mandarin-learning infants’ discrimination of tonal contrasts.

Study	Age group	Tonal contrasts	Designs	Results
**Tonal discrimination in perception**				
Mattock and Burnham (2006)	6- and 9-month olds	Thai rising tone vs. low-level tone	Head-turn procedure	Both age groups discriminated Thai tone contrasts.
Yeung et al. (2013)	4- and 9-month olds	Cantonese rising vs. mid-level tone	Preferential looking procedure	Both age groups showed stable tone discrimination.
Tsao (2017)	6–8-month olds and 10–12-month olds	Mandarin T1/3, T2/3 T2/4	Head-turn procedure	Infants improved sensitivity in Tone 1 vs. 3 contrast but not in Tone 2 vs. 3 and Tone 2 vs. 4 contrasts.
Cheng et al. (2013)	Newborns and 6-month olds	Mandarin T1/3 and T2/3	Oddball paradigm	Six-month olds demonstrated an adult-like cortical response to the T1 vs. T3 pair, but not to the T2 vs. T3 pair.
**Tonal discrimination in word learning**				
Tao and Xu (2013)	12-month olds	Mandarin T1/4, T2/4, T3/4	Preferential looking procedure	Infants distinguished the tonal contrasts in familiar word recognition.
Ma et al. (2017)	3-year olds	Mandarin T1/2, T1/3, T1/4, T2/3, T2/4, and T3/4.	Preferential looking procedure	Children only discriminated the T1/3 tonal contrast in familiar word recognition.
Singh et al. (2017)	3-year olds	Mandarin T1/4 and T2/3.	Preferential looking procedure	Children discriminated T1/4 tonal contrast but not T2/3 contrast in familiar word recognition.
Singh et al. (2016)	12- to 13-month olds and 17- to 18-month olds	Mandarin T1/3 and T2/3.	Habituation paradigm	The younger group did not interpret both the T1/3 and the 2/3 contrasts in the novel word learning, but the older group exploited both tonal contrasts.
Burnham et al. (2018)	17-month olds	Mandarin T1/2 and T2/4.	Habituation-switch paradigm	Infants discriminated the T1/2 but not the T2/4 tonal contrasts in novel word learning.

### Interpreting Tonal Contrasts in Word Learning

The aforementioned studies suggest that Mandarin-learning infants develop their language-specific categorical system of lexical tones before their first birthday. However, it is not clear whether children understand the phonemic function of tones, i.e., that tonal contrasts are meaningfully different in words, once they can discriminate them. As infants grow older, they narrow down or functionally reorganize their speech sound inventory based on the ambient language input (Saffran et al., 1996; Werker and Yeung, 2005). During their word learning, they map an entity to a potential word label that is picked up from their language-specific inventory (Namy and Waxman, 1998; Woodward and Hoyne, 1999). For example, they develop a sophisticated discrimination of their native phonemes (Werker and Fennell, 2004; Mani and Plunkett, 2007), and demonstrate sensitivity to word forms (e.g., phonotactics) of their native language as early as 12 months (MacKenzie et al., 2011, 2012). However, they also tend to use non-native cues (e.g., lexical tones) when the sounds are distinct (Hay et al., 2015). The remarkableness of cues may contribute to infants’ use of them in their word learning. In this context, it will be of great interest to explore questions such as when native tonal contrasts are incorporated into word learning, and whether the most distinctive Tone 1 and Tone 3 pair can be exploited at an early age (i.e., 12–14 months).

As Mandarin-learning children are more sensitive to the static vs. dynamic contrast than the dynamic vs. dynamic contrasts in tone perception, they have been found to be more likely to use the static vs. dynamic contrasts (rather than the dynamic vs. dynamic contrasts) in order to distinguish familiar words. Three-year-old Mandarin-speaking children may mistakenly fixate on familiar target objects even though the tones of the familiar words are mispronounced, but they will not increase their fixation to a familiar target when its static tone (Tone 1) is mispronounced into a dynamic tone (e.g., Tone 3 or Tone 4) (Ma et al., 2017; Singh et al., 2017).

Although Mandarin-speaking toddlers seem to be particularly sensitive to the static-dynamic contrasts in familiar word recognition, it is possible that these children are simply sensitive to the acoustic change. For example, for the high-frequency words such as *dog* or *ball,* 12-month-old Mandarin-learning infants have been found to prefer to look at the correct word-object pairs, rather than the ones whose tones are mispronounced by an experimenter (Tao et al., 2012; Tao and Xu, 2013). They may have simply detected the change in the words they are familiar with. It is thus still unclear whether infants consider tonal contrasts as phonemic cues in distinguishing word meanings. Seen in this way, it merits exploring whether younger language learners spontaneously use tonal cues to distinguish words in novel word learning.

In studies of novel word learning, it is generally found that native speakers of Mandarin do not interpret their native tonal contrasts until 17 months of age (Singh et al., 2016; Burnham et al., 2018). To cite an example, Burnham et al. (2018) habituated 17-month-old Mandarin-learning infants with two pairs of novel words and objects, and switched the two words in a test phase. In this situation, the infants had to decide which phonetic elements to use in order to distinguish words. The two words were only different in the lexical tone. A static vs. dynamic tonal switch (Tone 1 vs. Tone 2) and a dynamic vs. dynamic switch (Tone 2. vs. Tone 4) were tested, and infants were able to use the static vs. dynamic tonal switch, instead of the dynamic vs. dynamic switch, to distinguish novel words. A similar finding was reported by Singh et al. (2016), who revealed that native speakers of Mandarin did not interpret tonal contrasts until 17 months of age, although they distinguished them in a pre-lexical tone perception task at the age of 12–13 months.

Different with the tonal language infants who develop their use of native tonal cues as they grow, non-tonal language infants use non-native tonal cues in the word learning at an earlier stage, but tend to lose their tonal interpretation as they grow. For example, English-learning infants have been found to detect a tone switch in two word-object pairs at 14 months, but not 17 months (Hay et al., 2015). The younger group of 14 months may overgeneralize the function of pitch contours at the word level. However, they accept tonal variants as the same word when they gain more linguistic experience and understand the function of pitch in their native language at around 17–18 months.

Generally, previous findings reveal that at around 17 months, tonal language learners can interpret their native tonal cues in their novel word learning. Acoustic salience such as the static-dynamic discriminability may contribute to their interpretation (Ma et al., 2017; Singh et al., 2017; Burnham et al., 2018), as revealed in the tone perception studies (Cheng et al., 2013; Tsao, 2017). Evidence from non-tonal language learners indicates that at an early age, infants can use tonal cues based on acoustic salience (Hay et al., 2015). Given that Mandarin-learning infants show different degrees of sensitivity to different tonal contrasts, it would be necessary to provide further evidence of infants’ use of particular tones. As established, the Tone 1 and Tone 3 pair is the most perceptually salient tonal contrast. Mandarin-learning infants have been found to be sensitive to the Tone 1 vs. Tone 2 contrast during novel word discrimination at 17 months (Burnham et al., 2018). It is therefore likely that they can interpret the Tone 1 and Tone 3 pair in their word learning at an earlier stage.

In the present study, which used the same habituation-switch paradigm as Burnham and colleagues’ study, Mandarin monolinguals were presented with two words with two distinct lexical tones, Tone 1 vs. Tone 3 (/fāi/ vs. /fǎi/). Each token was paired with a visually distinct novel object in the habituation phase. The infants’ eye gaze was analyzed at the test phase, in which one of two word-object pairs was kept the same, while the other was changed. Two age groups (14 and 18 months) were chosen. At 14 months, infants are beginning to associate words to objects in the habituation-switch paradigm. Therefore, it seems reasonable to assume that infants can interpret a salient tonal contrast, such as Tone 1 vs. Tone 3, in their word learning. The age of 18 months was chosen for the other group to age-match the subjects in Singh et al. (2016) and Burnham et al. (2018), where infants were reported to succeed in their interpretation of tonal contrasts in novel word learning.

## Experiment 1

### Methods

#### Participants

Eighteen 14-month-old infants (9 females and 9 males, mean age = 14; 3, age range: 13; 21–15; 7) and twenty 18-month-old infants (10 females and 10 males, mean age = 18; 9, age range: 17; 5–19; 14) took part in the present study. The number of participants was determined based on previous studies (i.e., at least 16 infants per group; see Werker et al., 2002; Fennell and Waxman, 2010).

Data from an additional group of 21 infants were excluded from analysis for the following reasons: fussiness (two 14-month olds; six 18-month olds), non-recovery (five 14-month olds; four 18-month olds), max-out (one 14-month-old; two 18-month olds), and parental interference (one 18-month-old). Infants who did not look longer at the post-test trial than the test trials were considered to be fatigued and thus reported as “non-recovery.” Infants who watched all 20 habituation trials but did not reduce their looking time by 50% compared to their initial fixation were reported as “maxed out” (see “Procedure” section).

All infants were full term, had no apparent health problems, and been exposed only to Mandarin. All participants received a souvenir certification and transportation fee after the experiment.

#### Stimuli

The experiment was composed of four phases: a pre-test, a familiarization phase, a test phase, and a post-test. In the 15-s pre-test, a video clip was presented with a girl juggling three colorful balls. The video was accompanied by a repeated child-directed speech sound (e.g., “wa oh”). This clip was used to attract infants’ attention to the screen.

**Figure 2:**
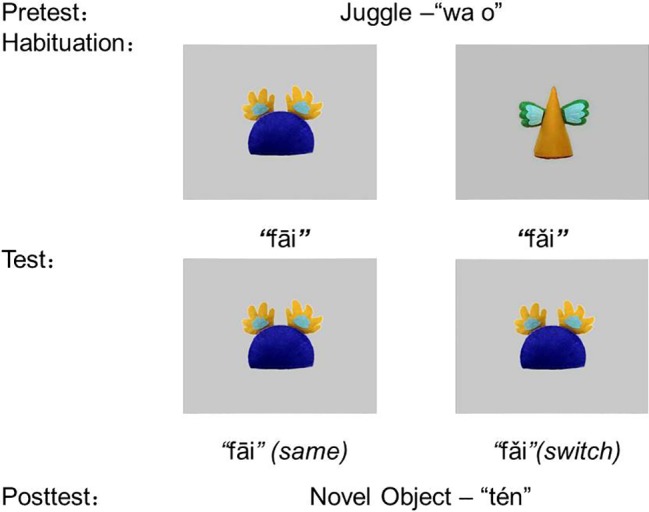
The procedure of the first experiment.

In the habituation and test phase, two types of 15-s clips were repeatedly played in a random order. In each clip, a novel object moved along a circle at an even speed. Two objects in each clip were markedly distinguishable in both shapes and colors. One of the objects was 14 cm high and 10 cm wide. It had a yellow cone body with two green and blue wing-like appendages. The other object was 11 cm high and 14 cm wide. It had a dark blue semicircle body with two yellow and blue hand-like appendages (see Figure 2).

**Figure 3:**
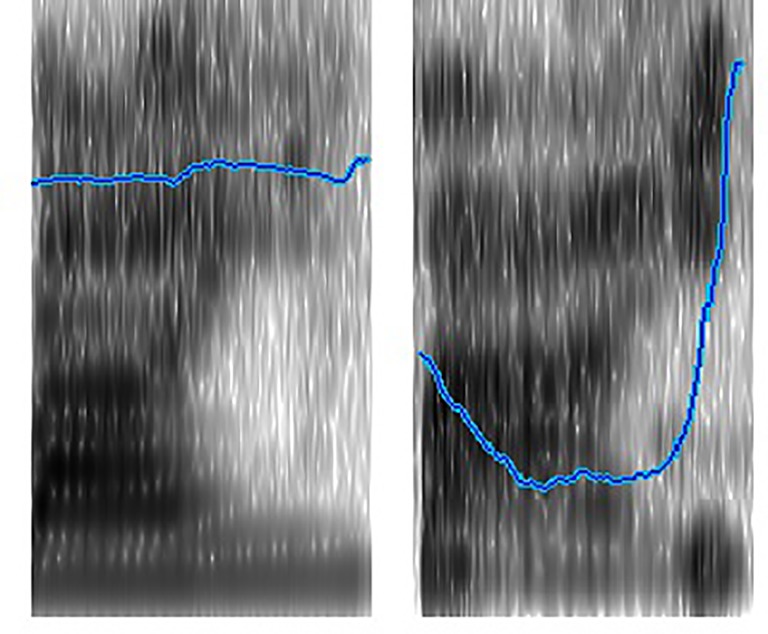
Spectrograms and pitch contours of the syllable /fai/ with first and third tones.

The video events were accompanied by two repeated tokens in child-directed speech. Two tokens were pre-recorded by a native Mandarin-speaking female in a soundproof room. They were the nonsense CVV Mandarin syllable with two distinct lexical tones (/fāi/ with the first tone and /fǎi/ with the third tone). The syllable was chosen because it is a non-word but it conforms to the phonotactic rule of Mandarin. Figure 3 shows the sound spectrograms of the two tonal words with their pitch variations. The token with the first tone had a duration of 959 ms. The first tone was level, starting at a frequency of 301 Hz, remaining stable and ending at 312 Hz. The token with the third tone had a duration of 936 ms. The third tone dipped, starting at 210 Hz, dropping to 145 Hz during the first 286 ms, remaining at this level for 430 ms, and then rising to 361 Hz over the remainder of the syllable (Figure 3).

In the post-test, a different novel object moved along a vertical path, accompanied by a novel utterance (tén). The post-test was designed to exclude those who were fatigued.

#### Apparatus

The experiment was carried out in a dim and soundproof room (12.3 m × 2.7 m), which was further divided into a testing area and a monitoring area by a dark blue curtain. An experimenter observed the infants’ online responses through a 4-cm hole in the curtain.

A table and a chair were placed in the testing area. A 17-inch video monitor embedded in a 55 cm × 45 cm black board was placed on the table. A Sony video camera was set up behind the black board to record infants’ eye movements from a 3-cm hole in the board, for the purpose of analysis and reliability coding.

During the test, the infants sat on their parents’ laps and watched video clips on the monitor. Their chair was approximately 70 cm away from the monitor. Parents were required to close their eyes during the test to avoid any disturbance to infants. If the parents opened their eyes and/or started talking, this was reported as parental interference in the “Participants” section. The audio stimuli were played at approximately 70 ± 5 dB measured by a sound level meter at the position of the infant’s face.

In the monitoring area, the experimenter, who was blind to the content of the stimuli, played the stimuli and recorded infants’ eye fixation through Habit 1.0 program in a Macintosh OS X system (Cohen et al., 2004).

#### Procedure

A short warm-up interaction was carried out to acquaint infants with the environment. Then the infants were brought into the testing area and the formal test started. To begin with, the pre-test (see Stimuli section) attracted infants’ attention to the monitor, followed by an attention-getter (a blue and white blinking cue with a “siren” sound). As soon as the infant orientated himself or herself toward the screen, the attention-getter stopped and the habituation phase began. This display of the pre-test and the attention-getter followed Zheng and colleagues’ study (Zheng et al., 2018).

The habituation phase consisted of five blocks. In each block, two word-object pairs were repeated twice in a random order, making up four trials in each block. A single trial ended once the infant looked away from the screen for more than 2 s, or if the 15-s clip was over. The looking time to a single trial is defined as the total play time of a video clip, including any looking-away time of less than 2 s. The habituation phase ended if the infant’s looking time across four consecutive trials decreased by 50% compared to their average looking time in the first block, or if a maximum of 20 trials were presented. The habituation criteria follow Werker et al. (1998).

Once infants reached the habituation criteria, two test trials, “same” and “switched” pairs, began. In the same test trial, the word-object pair was the same as in the habituation phase; in the switched test trial, either the word label or the object was switched with one in the other pair. The sample combinations of word-object pairs in the test phase are illustrated in Table 2.

**Table 2:** Sample combinations of word-object pairs in the test phase.

	Label switch		Object switch	
	Tone 1 switch	Tone 3 switch	Tone 1 switch	Tone 3 switch
Same trial	Object A – /fāi/	Object B – /fǎi/	Object A – /fāi/	Object B – /fǎi/
Switch trial	Object A – /fǎi/	Object B – /fāi/	Object B – /fāi/	Object A – /fǎi/

After two test trials, the post-test novel stimulus was presented to attract the infant’s attention again and exclude those who lost their concentration on the stimuli. As shown in the “Participants” section, if infants did not look longer at the post-test trial as compared to the test trials, the infants would be reported as “non-recovery” and thus excluded from analysis.

The combination of word-object pairs was counterbalanced. Half of the participants watched combination one (Object A-Word A, Object B-Word B), and the other watched combination two (Object A-Word B, Object B-Word A). Additionally, the switched pairs were counterbalanced. Half of the participants were presented with the Object A-Word B as the switched pair, and the others were shown the Object B-Word A pair. Half of the participants received a label switch, and the others received an object switch. Half of the participants received a Tone 1 switch, and the others received a Tone 3 switch (see Table 1). So, for the habituation pairs “object A – /fāi/ and object B – /fǎi/,” there are four types of combinations: two label switch and two object switch trials; two Tone 1 and two Tone 3 switch trials. The order of the two test trials was counterbalanced across infants: half of infants viewed the “switched” pair first, and others viewed the “same” pair first.

To sum up, the experiment had a 2 (test type: same vs. switch) by 2 (type of switch item: object switch vs. label switch) by 2 (type of switch tone: Tone 1 switch vs. Tone 3 switch) design. Test type was a within-subject manipulation, so a participant had both the same and the switch conditions, while type of switch item and type of switch tone were between-subject manipulations, so a participant had either the label switch or the object switch condition, and, at the same time, either a Tone 1 switch or a Tone 3 switch condition (see Table 1).

Before the analyses, a second coder who was blind to the experimental content recoded a random sample of 25% of the whole experimental session, and the inter-rater reliability was sufficiently high (*r* = 0.95).

### Results

Firstly, in order to examine whether perceptual attraction was consistent across demographic populations, the infants’ performances in the habituation phase and the pre- and post-test trials were compared between two age groups. The looking time in the habituation phase, *F*(1, 36) = 1.354, *p* = 0.252,$\eta_{p}^{2}$ = 0.036, and the number of habituation trials, *F*(1, 36) = 2.166, *p* = 0.150, $\eta_{p}^{2}$ = 0.057, did not differ between the two age groups. The infants’ looking time in the first block, *F*(1, 36) = 0.519, *p* = 0.476,$\eta_{p}^{2}$ = 0.014, the last block, *F*(1, 36) = 0.021, *p* = 0.887,$\eta_{p}^{2}$ = 0.001, and the drop in their looking time (the ratio of looking time to the last block over that to the first block), *F*(1, 36) = 0.097, *p* = 0.757,$\eta_{p}^{2}$ = 0.003, did not differ between the two age groups, suggesting that no systematic bias in attention was found between the groups. The looking time in the pre- and post-test trials did not differ, *F*(1, 36) = 1.595, *p* = 0.215, $\eta_{p}^{2}$ = 0.042, between two age groups, *F*(1, 36) = 2.182, *p* = 0.148, $\eta_{p}^{2}$ = 0.057, and no significant pre-/post-trial by age group interaction was found, *F*(1, 36) = 1.043, *p* = 0.314,$\eta_{p}^{2}$ = 0.028, suggesting that attention did not significantly reduce over time.

The main purpose of this study is to explore infants’ word-object association, specifically posing the question of whether infants would be dishabituated to the switched pair. A mixed ANOVA was carried out with a full model 2(age: 14 vs. 18 m) by 2(test type: same vs. switch) by 2(gender: male vs. female) by 2(type of switch item: object switch vs. label switch) by 2 (type of switch tone: Tone 1 switch vs. Tone 3 switch) design with the looking time to the test trials as the dependent variable. But the following ANOVA and *post hoc* analyses collapsed across gender, type of switch item, and switch tone, because no main effect or interactions were revealed. There was a marginal effect of test type, *F*(1, 36) = 3.951, *p* = 0.054, $\eta_{p}^{2}$ = 0.099, revealing that infants have a longer looking time to the switched trial than the same trial. There was also a marginal effect of age, *F*(1, 36) = 4.091, *p* = 0.051, $\eta_{p}^{2}$ = 0.102, showing that the 14-month-olds have an overall longer looking time to the test trials than the 18-month olds. However, the interaction between the two variables is not significant, *F*(1, 36) = 1.519, *p* = 0.226, $\eta_{p}^{2}$ = 0.040, implying that sensitivity to tonal cues is not significantly different between the two age groups.

**Figure 4:**
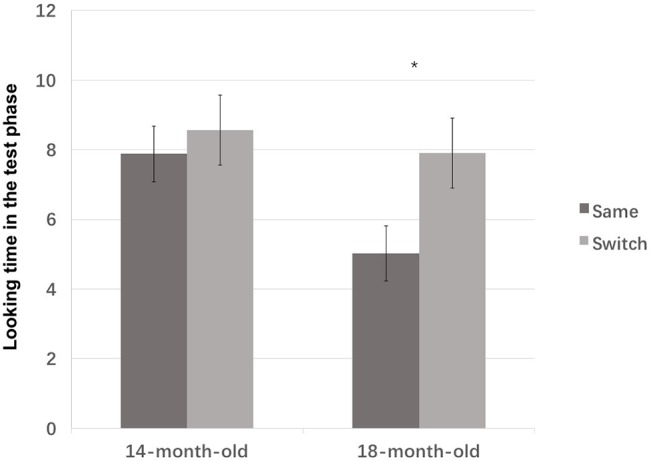
The average fixation to the video clips during the test phase for 14- and 18-month-old infants in Experiment 1. Error bars represent standard errors. *p < 0.05.

As indicated in the “Introduction” section, the younger group was chosen because they represented a transitional learning stage, and the older group was chosen in order to test the previous findings that infants can interpret tonal cues in novel word learning. Therefore, a planned comparison with Bonferroni corrections was conducted to analyze the switched effect in each age group. No difference was found between the same (*M* = 7.88, SD = 4.35) and the switched trials (*M* = 8.56, SD = 4.05), *F*(1, 36) = 0.271, *p* = 0.606, $\eta_{p}^{2}$ = 0.007 (Figure 4) in the 14-month-old group, failing to show that the young group could attend to tonal information during their word-object association. But an effect of the test type was found in the 18-month-old group due to a significantly longer looking time to the switched trial (*M* = 7.91, SD = 4.44) than the same trial (*M* = 5.01, SD = 2.14), *F*(1, 18) = 5.473, *p* = 0.025, $\eta_{p}^{2}$ = 0.132 (Figure 4), confirming that the older group was able to use tonal information during their word-object association. The average fixation to the video clips in each test trial was further illustrated by the age group in Figure 4.

**Figure 5:**
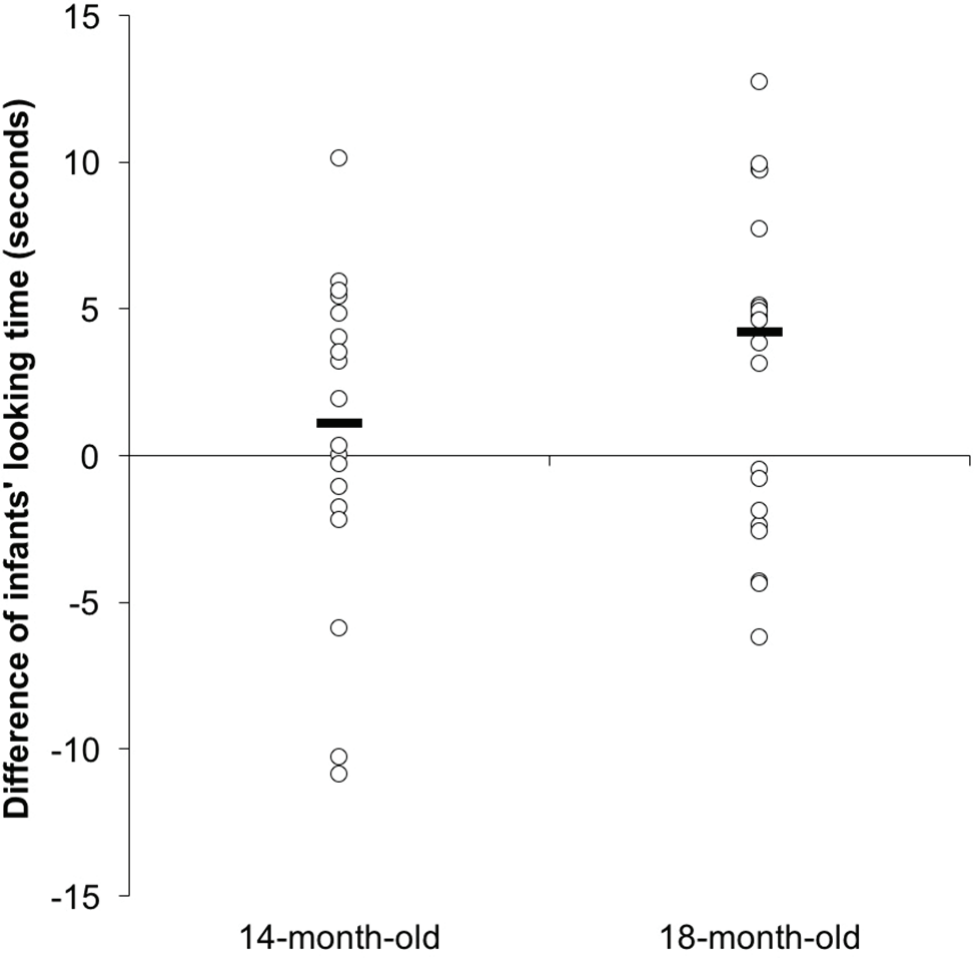
The scatterplots of difference of infants’ looking time to both test trails. The scatterplots show the difference of infants’ looking time to two test trials in each individual. The difference was calculated through subtracting the looking time to the “same” trial from that of the “switched” trial. So, a positive value represents that the individual was dishabituated to the switched trial. The bold lines show the median of the difference.

A non-parametric test was also conducted and showed a similar result. A Wilcoxon signed-rank test showed that 14-month olds looked at both test trials for a comparable length of time, *z* = −0.734, *p* = 0.463. Ten of the 18 infants showed a longer looking time to the switched trial than to the same trial, seven showed a reverse pattern, and one showed an equal looking time to both tests. However, the 18-month olds showed a significantly longer looking time to the switched trial than to the same trial, *z* = −2.165, *p* = 0.03. Twelve out of 20 infants showed a longer looking time to the switched trials, while eight showed a reverse pattern. Figure 5 illustrates the difference in looking time to two test trials in each individual.

#### Summary for the Results

Experiment 1 suggests that only the 18-month-old children can associate two tonal words with different objects. However, before this conclusion can be confirmed, the word association ability of the Mandarin participants should be examined. Few studies, so far, have examined Mandarin-learning infants with the word-object switch habituation task. Furthermore, it is not clear whether 14-month-old Mandarin infants can establish word-object association even if the two words are distinctive enough. Therefore, the next experiment would use two distinct Mandarin syllables (fāi vs. bǒu) to test their associative learning ability and set up a baseline for the first experiment. The following task would lead to a comparison of Mandarin-learning infants’ tone association with that of their English contemporaries.

## Experiment 2

As in the first experiment, the habituation-switch task was manipulated by switching either the words or the tones of two word-object pairs for groups of infants aged 14 and 18 months. Two distinct audio stimuli (fāi vs. bǒu), non-words but conforming to the phonotactic form of Mandarin, were used. The first and third tones were selected in order to match the tonal cues in the first experiment.

### Methods

#### Participants

Thirty-eight infants participated in the present study. They were divided into two age groups: 14 and 18 months. Eighteen of the participants were 14 months old (9 females and 9 males mean age = 14; 5; age range = 13; 20–14; 27) and 20 of them were 18 months old (10 females and 10 males mean age = 18; 1; age range = 17; 7–19; 2). All infants were full-term infants and had no apparent health problems.

Data from an additional group of 21 infants was excluded from analysis for the following reasons, respectively: fussiness (two 14-month olds; two 18-month olds), non-recovery (six 14-month olds; five 18-month olds), max-out (four 14-month olds; one 18-month olds), and experimenter error (one 18-month-old). The study was approved by the Institutional Review Board of the University. Written informed consent was obtained from the parents of the participants before the experiments. All children received a souvenir certification and transportation fee after the experiment.

#### Stimuli and Procedure

The second experiment was carried out in the same area as the first experiment, with the same experimental setup. The only difference existed in the auditory stimuli (/fāi/ vs. /bǒu/), which are different in both syllable and tone.

### Results

Firstly, the infants’ performance in the habituation phase and the pre- and post-test trials was compared across the two age groups. The looking time in the habituation phase, *F*(1, 36) = 2.038, *p* = 0.162,$\eta_{p}^{2}$ = 0.054, and the number of habituation trials, *F*(1, 36) = 0.783, *p* = 0.382, $\eta_{p}^{2}$ = 0.021, did not differ between the two age groups. The infants’ looking time in the first block, *F*(1, 36) = 3.268, *p* = 0.079,$\eta_{p}^{2}$ = 0.083, the last block, *F*(1, 36) = 0.005, *p* = 0.945,$\eta_{p}^{2}$ = 0.000, and the drop in their looking time, *F*(1, 36) = 1.446, *p* = 0.237,$\eta_{p}^{2}$ = 0.139, did not differ between the two age groups, suggesting that there was no systematic bias in attention between the groups. Similarly, the looking time in the pre- and post-test trials did not differ, *F*(1, 36) = 0.890, *p* = 0.352, $\eta_{p}^{2}$ = 0.024, between two age groups, *F*(1, 36) = 0.768, *p* = 0.387, $\eta_{p}^{2}$ = 0.021, and no significant pre-/post-trial by group interaction was found, *F*(1, 36) = 0.081, *p* = 0.778,$\eta_{p}^{2}$ = 0.002, suggesting that attention did not significantly reduce over time.

In the main analysis, a mixed ANOVA was carried out with a full model 2(age: 14 vs. 18 m) by 2(test type: same vs. switch) by 2(gender: male vs. female) by 2(type of switch item: object switch vs. label switch) by 2(type of switch label: /fāi/ switch vs. /bǒu/ switch) design with the looking time to the test trials as the dependent variable. But the following ANOVA and *post hoc* analyses collapsed across gender, type of switch item, and switch label, because no main effect or interactions were revealed. A significant main effect of the test type revealed that infants looked longer to the switched trials (*M* = 8.84, SD = 4.49) than to the same test (*M* = 6.42, SD = 3.98), *F*(1, 36) = 9.720, *p* = 0.004, *η*^2^ = 0.213. No other significant main or interaction effects were found.

**Figure 6:**
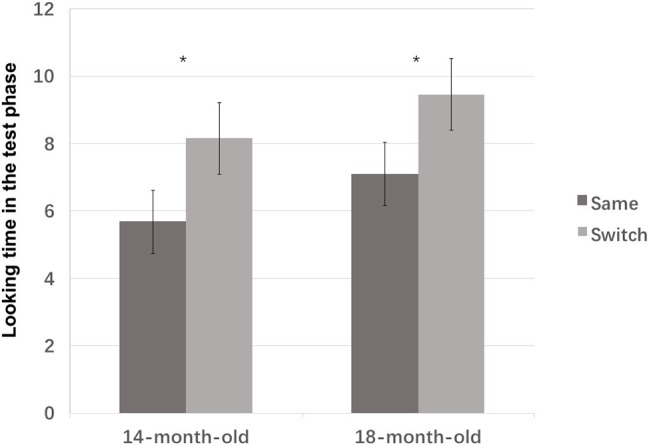
The looking time during the test phase for 14- and 18-month-old infants in Experiment 2. Error bars represent standard errors. *p < 0.05.

A planned comparison with Bonferroni corrections revealed that the test type had an effect in both age groups. Infants had a significantly longer looking time to the switched trial than the same trial [14-month olds: *F*(1, 36) = 4.855, *p* = 0.034, $\eta_{p}^{2}$ = 0.119; 18-month olds, *F*(1, 36) = 4.873, *p* = 0.034, $\eta_{p}^{2}$ = 0.119], indicating that both age groups were able to use established word-object association (Figure 6).

**Figure 7:**
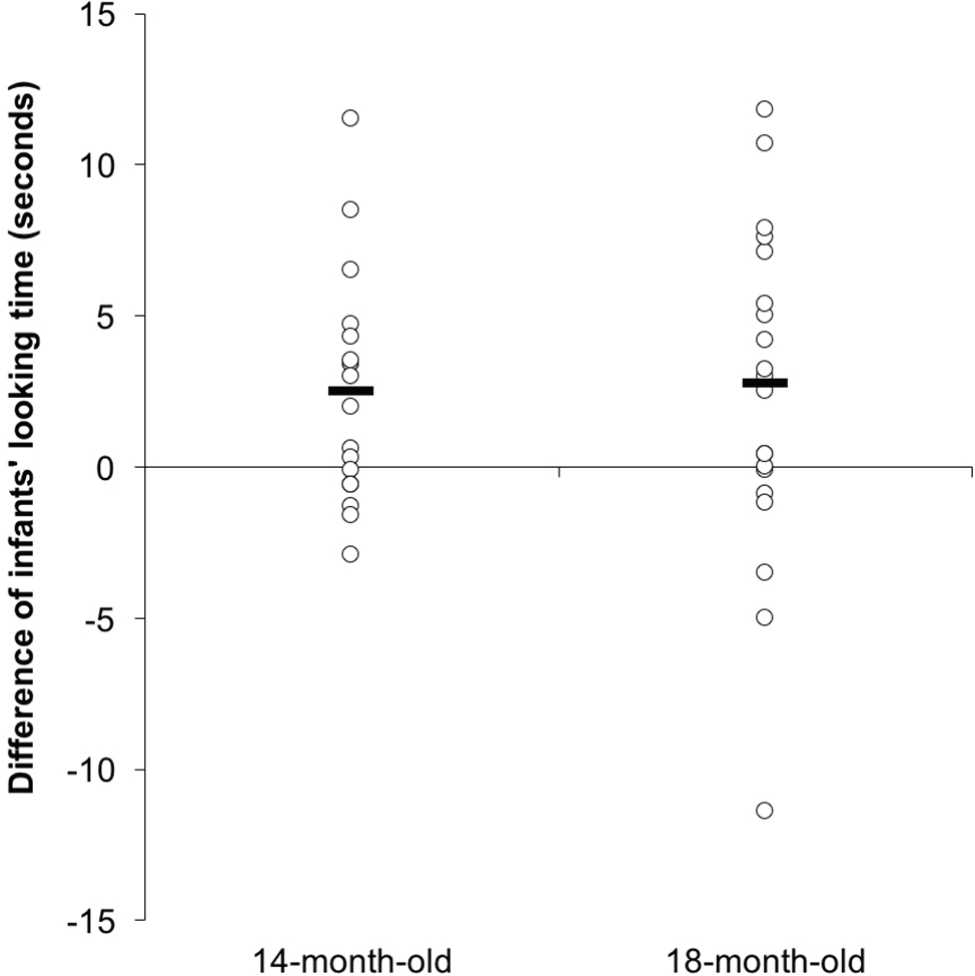
The scatterplots of difference of infants’ looking time to both test trails.

In the non-parametric comparisons, the Wilcoxon signed-rank test showed that 14-month olds had longer looking times to the switched trial than to the same test, *z* = −2.375, *p* = 0.018. Twelve out of the eighteen 14-month-old infants showed a longer looking time to the switched trial than the same trial, while only six of them showed a reverse pattern, while 18-month olds showed the same pattern, *z* = −1.872, *p* = 0.061. Thirteen out of the 20 infants showed a longer looking time to the switched trial than the same trial, six of them showed a reverse pattern, and one showed an equal looking time to both tests. Figure 7 illustrates the scatterplots of the difference between the infants’ looking time in the two test trials.

In sum, both 14- and 18-month-old Mandarin-learning infants demonstrated word-object association ability, as their English contemporaries did in previous studies (e.g., Werker et al., 1998, 2002).

## General Discussion

The present study used two sets of habituation-switch experiments to explore Mandarin-learning infants’ word-object association. The first experiment particularly tested the infants’ discrimination of the most significant tonal contrast, the Tone 1 and Tone 3 pair, in word-object association, and revealed a marginal effect on their detection of the switched tones across two age groups. However, the planned *post hoc* comparisons demonstrated that the Mandarin-learning infants failed to establish tonal word associations at 14 months but succeeded at 18 months of age, even though the effect of the test trial type did not interact with age.

The ability of 18-month-old infants to interpret the Tone 1 and Tone 3 contrast during their novel word learning shown in this experiment is consistent with Burnham et al.’s (2018) finding, which showed that another static vs. dynamic tonal contrast (e.g., the Tone 1 and Tone 2 pair) could be interpreted by 17-month-old infants during their novel word learning. Burnham and colleagues’ study found that infants could interpret the static vs. dynamic tonal contrast (e.g., the Tone 1 and Tone 2 pair), but not the dynamic vs. dynamic contrast (e.g., the Tone 2 and Tone 4 pair). This indicates that the static vs. dynamic tonal contrasts are more distinctive than the dynamic vs. dynamic contrasts, and such discriminability of tonal contrasts contributes to the older infants’ (i.e., 17-month olds) interpretation of tonal cues. In this context, the present study provides well-needed supplemental data to confirm that the particular static vs. dynamic contrast (e.g., the Tone 1 and Tone 3 pair) can be also interpreted at the age of 18 months.

Our second experiment indicated that infants could associate distinct words with different objects as early as 14 months; therefore, the failure of the younger group in the first experiment was probably not caused by an inability to form word-object associations. In fact, studies on phonetic segments (Stager and Werker, 1997; Werker and Fennell, 2004) also have reported that infants of this age cannot exploit subtle phonemic contrasts during the word learning process at 14 months. Stager and Werker (1997) showed that 14-month-old English-learning infants could detect a switch of two labels that were markedly different, but failed to do so when the word labels were minimal pairs (e.g., /dih/ vs. /bih/). This might be explained by infants’ limited cognitive resources for attending to more subtle contrasts (Werker and Fennell, 2004). In a familiar word recognition task where less cognitive resource is needed, 12-month-old Mandarin-learning infants looked at familiar objects less frequently when the tone was mispronounced than when the word label was correctly pronounced (Tao et al., 2012; Tao and Xu, 2013). In recognition studies, infants have been equipped with a lexical representation of a word token, so their task is to compare a new token with the prototypical word individually. Their sensitivity to the acoustic distinction between the new word and the original one should suffice for tonal word recognition. Accordingly, infants’ performance in word learning relies highly on both language knowledge and the particular cognitive ability required by specific tasks (Curtin and Werker, 2018).

Based on the novel word learning task, the first experiment may imply that the development of a mature phonemic system of native lexical tones has not been completed in 14-month-old Mandarin-learning infants. During their novel word learning, infants have to decide which phonetic elements can be used to designate the meaning of a word when they associated the word with an entity. In previous studies, infants may rely on low-level cues, i.e., acoustic salience, in their word learning at 14 months, even though the cues do not convey lexical meanings in their native language (Hay et al., 2015). Similarly, none of the previous studies provided evidence that Mandarin-learning infants interpreted lexical tones in their novel word learning at 14 months (see, for example, Singh et al., 2016). These findings may imply that Mandarin-learning infants have not been successful in integrating their knowledge of phonemic minimum pairs with lexical tones at 14 months of age.

Infants’ interpretation of lexical tones may also be influenced by their developing knowledge of intonation. For native speakers of tone languages, tones sometimes share their variation of pitch contours with intonations. As reviewed in the section “Introduction,” pitch contours carry either lexical or pragmatic meanings for tonal language speakers. For example, the rising contour in Mandarin can be perceived as either the lexical Tone 2 or the intonation category for questions (Bolinger, 1958). Seen this way, children of tone languages need to decide whether a pitch contour is meaningful at a word or an utterance level. They may mistakenly perceive pitch contours of lexical tones at the word level as belonging to intonational contrasts at the utterance level (i.e., as discussed by Burnham et al., 2018). Their word recognition may be influenced by the dissociated pitch context where a lexical tone (e.g., rising) is produced in a conflicting intonation (e.g., falling; Singh and Chee, 2016). For non-tonal language learning infants, although pitch variations only involve pragmatic implications at an utterance level, they may use tones to distinguish word meanings (Hay et al., 2015). In particular, their sensitivity to tonal contrasts rebounds at their second year of life, because their increasing experience of pitch variations in intonation may facilitate their tone perception (Liu and Kager, 2014). In this light, although Mandarin-learning infants can perceptually distinguish lexical tonal contrasts before their first birthday, it would still be hard for them to differentiate the lexical function of tones from the pragmatic implication of intonations.

It may be questioned whether the complex phonetic property of Tone 3 also contributes to the failure of the younger group in our study. Tone 3 is the most difficult tone to produce, not only because it involves double contours *via* lowering and rising the f0 (see Figure 1), but also because Tone 3 sandhi is the most common tonal sandhi in Mandarin (Li and Thompson, 1977). Tone 3 sandhi describes how Tone 3 changes to a rising tone if it precedes another dipping tone, and changes to a falling tone if it precedes any other tones. Due to its variation, Tone 3 is less frequently perceived than the other three tones. Seen in this way, infants may not establish a precise phonemic system for Tone 3 due to its variation in language input and therefore do not consider it as a cue to distinguish word meanings. Having said that, the complexity of Tone 3 may not have influenced the finding of the present study, given that there was no main effect of the type of switch tone, and any interaction with it. If the infants had linked either of the two word-object pairs, they would have been dishabituated to the relevant switched tone condition in which the tone of that pair was changed.

In sum, the present study not only explored Mandarin infants’ tonal interpretation, but also tested their basic learning ability using two distinctive words. Using the latter as a baseline, we found that tonal word learning occurs later than distinctive word learning. The present study did not generate evidence in support of the claim that 14-month-old infants can use the salient Tone 1 and Tone 3 contrast to distinguish novel words. Given that both lexical tones and phonetic segments are used to distinguish meanings, future studies should compare infants’ performance in tonal word learning with their discrimination of minimal pairs (e.g., /bou/ vs. /pou/) and could explore whether any pragmatic interpretation of Tone 1, Tone 3, or both, may influence infants’ word learning.

## Ethics Statement

We certify that the research adhered to the ethical principles of the American Psychological Association [APA (2010)]. Written informed consents were obtained from the parents of the participants before the experiments. Participants who are unwilling to continue the experiment can withdraw from the study at any time. The data in this experiment will be treated confidentially and used only for scientific purposes. This study was carried out in accordance with the recommendations of “Institutional Review Board of the School of Psychological and Cognitive Sciences, Peking University” with written informed consent from all subjects. All subjects gave written informed consent in accordance with the Declaration of Helsinki. The protocol was approved by the “Institutional Review Board of the School of Psychological and Cognitive Sciences, Peking University.”

## Author Contributions

XZ performed data collection and analysis and drafted the manuscript. XM and YJ provided critical revisions. YJ edited the manuscript. All authors developed the study concept, contributed to the study design, and approved the final version of the manuscript for submission.

### Conflict of Interest Statement

The authors declare that the research was conducted in the absence of any commercial or financial relationships that could be construed as a potential conflict of interest.

## Footnotes

^1^Please refer to Figure 1 for the f0 contours of four Mandarin tones.
